# Patient Interval and Associated Factors in the Diagnostic Journey of Oral Cancer: A Hospital-Based Cross-Sectional Study from Kerala, India

**DOI:** 10.31557/APJCP.2021.22.10.3143

**Published:** 2021-10

**Authors:** Phinse Mappalakayil Philip, Srinivasan Kannan

**Affiliations:** *Achutha Menon Centre for Health Science Studies, Sree Chitra Tirunal Institute for Medical Sciences and Technology, Trivandrum, Kerala, India. *

**Keywords:** Delay in presentation, Patient delay, Early diagnosis, Symptom awareness, Aarhus statement

## Abstract

**Background:**

The incidence of oral cancer is increasing in south-central Asia. Though it can be detected early, most cases were reported in late stages, resulting in a poor prognosis. Reducing the patient interval will facilitate early diagnosis and better disease survival. The paucity of research on the patient interval in oral cancer has limited our ability to design and evaluate programs for early diagnosis.

**Methods:**

The study was conducted to identify the duration of patient interval and associated factors in oral cancer. Patients with oral cancer reporting at a tertiary cancer center during the study period were interviewed using validated data collection tools. The ‘Aarhus statement’ guidelines were followed in designing and reporting the study.

**Results:**

Among the 261 participants, 54% reported a patient interval of more than 90 days. The median (IQR) patient interval was 92 (38-168) days. In the multivariate binary logistic regression model, those who approached healthcare facilities due to pain (OR, 8.3, 95% CI, 2.9 to 23.4) were more likely to have a patient interval of more than 90 days over those who came due to insistence by family. Smoking status (Current smoker vs. never smoker) at the time of diagnosis (OR, 2.518, 95% CI, 1.3 to 4.7), Stage of cancer (late vs. early) of participants (OR, 2.62, 95% CI, 1.3 to 5.2), and time of travel (>30 minutes vs. ≤ 10 minutes) to health care facility (OR 5.8, 95% CI, 1.6 to 21.7) were the other significant predictors for the patient interval of more than 90 days.

**Conclusion:**

Patient interval in oral cancer can be reduced by improving symptom awareness, abstinence from tobacco use, and facilitating access to health care facilities. The double burden of tobacco use in oral cancer, as it increases the risk of disease occurrence and delays symptom presentation, needs serious policy considerations in the context of cancer prevention.

## Introduction

Oral cancer is the 16^th^ most common cancer in the world with 3,77,713 cases reported in the year 2020. Of these, 84% were from Asia and Europe (Sung et al., 2021). It is the second most common cancer in India and this contributes to more than one-third (36%) of total oral cancer incidence in the world (Ferlay et al., 2020). An analysis of the data from 29 cancer registries in India reported a general uptrend in the incidence of oral cancer (Sharma et al., 2018). The risk factors for oral cancer are well known and desisting from those habits is a time-tested primary prevention strategy. Several favorable factors exist for its early diagnosis. The ease of access to the oral cavity for physical examination is one among them (Shrestha and Maharjan, 2020). Dental and medical primary care providers and trained health workers can identify oral cancer through oral visual examination. At times, many are preceded by precancerous lesions that can be detected and managed to prevent them from progressing to oral cancer (Sankaranarayanan et al., 2015). Despite all these favorable factors, oral cancer is continuously been reported and diagnosed in late stages leading to poor treatment outcomes (Güneri and Epstein, 2014). 

An important factor associated with an advanced stage at diagnosis is the patient delay (Alahapperuma and Fernando, 2017). Early diagnosis has a significant impact on the disease outcome and survival (World Health Organization, 2017). The five-year survival rate of early-stage oral cancer is around 80%, while that of late-stage disease is nearly 20%. Moreover, 50% of oral cancer cases are presented in very late stages. So emphasis should be given to early diagnosis to improve survival rates (van der Waal, 2013). If a patient takes more than three months to meet a health care professional after recognizing a symptom suggestive of cancer is considered as undue delay (Pack and Gallo, 1938). Several circumstances and factors are responsible for this delayed presentation (Philip and Kannan, 2019). It is inappropriate to blame the patient for the same. Considering this, the World Health Organization (WHO) has suggested using the term “Patient interval” instead of “Patient delay” (World Health Organization, 2017). Though studies were conducted to measure this interval and to know about the contributing factors, they did not have uniformity in their approaches and definitions making comparisons across such studies difficult. Considering this, a consensus workshop of early cancer researchers was convened and developed “Aarhus statement” consisting of definitions and recommendations for early cancer diagnosis research (Weller et al., 2012). There is a paucity of early cancer diagnosis research studies that focus on oral cancer (Philip and Kannan, 2019). This creates a serious knowledge gap for guiding further research as well as for planning interventions to address the delay in reporting symptoms suggestive of oral cancer. Moreover, region-specific data on the magnitude of patient interval and associated factors are necessary for planning tailored programs. The objective of the present study was to identify the duration of patient interval and its contributing factors among the oral cancer patients reporting at a tertiary cancer center in Kerala, India.

## Materials and Methods

*Methodology*

This hospital-based cross-sectional study was conducted using validated tools for measuring patient interval and identifying contributing factors. The estimated dates of events provided by patients were used for calculating pseudo exact dates with the help of a protocol developed by Neal (2014). We followed the guidelines given in the Aarhus Statement for designing the study (Weller et al., 2012). The study was conducted at a tertiary care hospital in Northern Kerala from December 2019 to August 2020. Each consecutive oral cancer patient who reported at the institution during the study period and who met the eligibility criteria and consented to enrol was included in the study until the required sample size was reached. The newly registered patients with malignant neoplasm of the oral cavity (ICD code C00 to C06) were included in the study. Patients having a recurrence, patients with other cancers, patients who were diagnosed through routine cancer surveillance programs, patients who had completed treatment and registered only for follow up and patients who were not willing to participate in the study were excluded. Details about sample size, sample selection, variables, data sources, data collection methods were given elsewhere (Philip and Kannan, 2019). 

The study received approval from the institutional Ethics Committee and Technical Advisory Committee of the institution where the principal investigator is a research scholar (SCT/IEC/1388/JUNE-2019). Ethics committee approval was also obtained from the institution where the study was conducted (1617/IRB-IEC/13/MCC/13-05-2019/5). We took every effort to minimize recall bias in our study. We corroborated the information provided by the participants with their medical records and referral letters. We used validated instruments for data collection and a protocol was used for validating the estimated dates provided by the participants. The main dependent variable in the study, patient interval, was measured in days. Participants were then categorized into two groups based on the duration of the patient interval. The first group consists of participants having a patient interval less than or equal to 90 days. The second group includes those participants having a patient interval of more than 90 days. This categorization was based on the arbitrary definition of undue delay as more than three months (Andersen et al., 2009). Moreover, a patient interval of more than three months was associated with lower survival rates (Richards et al., 1999). The study by Akram (2014) also considered a patient interval of more than three months as a delayed presentation.

The collected data was cleaned, rechecked, and prepared for analysis. Univariate analysis was performed to describe the data and to identify important covariates that could affect the patient interval. Continuous variables which are normally distributed were summarized using mean (standard deviation) and non-normally distributed ones were reported by median (Interquartile range). Patient interval data are usually positively skewed and reported with the median (Dobson et al., 2014). Categorical variables were analyzed using contingency tables. Chi-square statistics or Fisher’s exact test, when appropriate, were used to test the significance of relationships. Also, we collapsed those categories with low cell counts for a sensible analysis and meaningful result. 

Variables that had a significant relationship with patient interval and variables of known importance in the existing literature were planned to include in the binary logistic regression analysis for getting a model of the relationship between them and the patient interval. First, logistic regression was done with the single covariate of interest, for selecting the most influencing variables that are to be included in the final model. All selected independent variables (with p < 0.25) were added in the binary regression model in various combinations to get a best-fitting model. 

## Results

Data from 261 oral cancer patients reported at a tertiary cancer center in Kerala were collected and analyzed. The mean age of the participants was 60.76 years (SD=12.27). The male-to-female ratio was 2.4:1. Half of the participants were aged between 53 and 70 years. The mean age of female participants (64.45±10.86) was higher than that of males (59.25±12.56) and the difference was statistically significant (p=0.002). The majority of the participants (94.3%) had education up to the high school level and were daily wagers (64.4%) or farmers (13.8%). The median (Inter Quartile Range) patient interval was 92 (37.50-167.50) days [males-92(44.50-158.00), females- 90 (31.25-179.50) days]. The proportion of participants with patient interval duration equal to or less than 90 days was 46% and those with duration more than 90 days was 54%. 

The association of patient interval with socio-demographic and habit-related factors was analyzed (Table 1. Selected Socio-Demographic and Habit Related Factors Associated with the Patient interval in Oral Cancer Patients). After recognizing the cancer-related symptoms, 60.8% (n=62) of smokers, 46% (n=58) of betel quid users, 56.5% (n=13) of pan masala users, and 22.8% (n=28) of alcohol users either reduced or stopped their habits. It was observed that, those participants who continued or increased their habit of chewing tobacco after symptom identification were more likely to have a patient interval of more than 90 days compared to those who decreased or quit the habit (OR, 2.76 95% CI, 1.3 to 5.7). Compared to persons without any habits, those with habits had higher chances of getting a patient interval of more than 90 days (OR, 2.74; 95% CI, 1.1 to 6.6). 

Financial factors such as, “availability of government or private insurance”, “having another earning member in the family”, “dependence on others for meeting their financial needs”, and “having financial liabilities” were not significantly associated with the patient interval. 

Information on participants’ health-related practices (Table 2: Association of various factors with patient interval in oral cancer patients) and the barriers towards help-seeking were collected (Table 3: Endorsement of barriers to help-seeking and Patient interval in oral cancer patients). 

Discussion interval is the time taken by the participant for discussing the problems in their oral cavity with someone in the family or among his or her social circle, before meeting a health care professional. Among the participants (n=261), 69% discussed their oral problems, with a median (Interquartile range) discussion interval of 14 (7-30) days. Persons with whom these participants first discussed their symptoms were spouses (45%), sons (18.3%), daughters (12.2%), or friends (10%). Participants with a discussion interval of more than 30 days were more likely to have a patient interval of more than 90 days (OR, 7.85; 95% CI, 4.03 to 15.29) compared to those with less than 30 days of discussion interval.

A binary logistic regression was performed to analyze the factors that predict the patient interval. The patient interval of “more than 90 days” and “less than or equal to 90 days” were the outcomes. A final model was obtained with non-significant Hosmer and Lemeshow goodness of fit (*χ*^2^ (8) = 4.977, p=.760), indicating that the model adequately describes the data. The predictor variables identified were “Reason for meeting HCP for current problem in the oral cavity”, “Distance to the nearest healthcare facility”, “Cancer stage”, “worry about what the doctor might find out”, and “Tobacco smoking status at the time of symptom recognition” (Table 4: Summary of Binary Logistic Regression Analysis for Variables Predicting Patient interval). 

**Table 1:** Selected Socio-Demographic and Habit Related Factors Associated with the Patient interval in Oral Cancer Patients (n= 261)

Variable	≤ 90 days n (%)	> 90 days n (%)	Total n (%)	Chi-square p value
Age				
Below 60 yrs	63 (52.5)	73 (51.8)	136 (52.1)	0.907
Above 60 yrs	57 (47.5)	68 (48.2)	125 (47.9)	
Sex				
Female	39 (32.5)	37 (26.2)	76 (29.1)	0.267
Male	81 (67.5)	104 (73.8)	185 (70.9)	
Caste				
General class	25 (20.8)	22 (15.6)	47 (18.0)	
Other Backward class	72 (60.0)	95 (67.4)	167 (64.0)	0.435
Scheduled Caste	11 ( 9. 2)	8 ( 5. 7)	19 ( 7. 3)	
Scheduled Tribe	12 (10.0)	16 (11.3)	28 (10.7)	
House type				
Pucca	68 (56.7)	58 (41.1)	126 (48.3)	.012*
Semi pucca/Kutcha	52 (43.3)	83 (58.9)	135 (51.7)	
Tobacco smoking				
Current smoker	38 (31.7)	63 (44.7)	101 (38.7)	
Former smoker	19 (15.8)	11 ( 7. 8)	30 (11.5)	.033*
Never smoker	63 (52.5)	67 (47.5)	130 (49.8)	
Betel quid chewing				
Current user	56 (46.7)	70 (49.6)	126 (48.3)	
Former- user	9 (7.5)	13 (9.2)	22 ( 8. 4)	0.713
Non- user	55 (45.8)	58 (41.1)	113 (43.3)	
Alcohol usage				
Current user	51 (42.5)	70 (49.6)	121 (46.4)	
Former- user	5 (4.2)	6 (4.3)	11 (4.2)	0.496
Non –user	64 (53.3)	65 (46.1)	129 (49.4)	
Total	120 (46.0)	141 (54.0)	261 (100)	

*, p value less than 0.05

**Table 2:** Association of Various Factors with Patient Interval in Oral Cancer Patients (n= 261)

Variables	≤ 90 days n (%)	> 90 days n (%)	Unadjusted OR (95% CI)
Access and Pattern of Healthcare related factors			
Pattern of medical consultation			
Health screening at intervals	37 (30.8)	21 (14.9)	Reference
Medical consultation for illness	53 (44.2)	50 (35.5)	1.662 (0.9-3.2)
Urgent medical care only	30 (25.0)	70 (49.6)	4.111 (2.1-8.2)
Pattern of dental consultation			
Dental screening at intervals	10 (8.3)	4 (2.8)	Reference
Dental consultation for illness	54 (45.0)	31 (22.0)	1.435 (0.4-4.9)
Urgent dental care only	56 (46.7)	106 (75.20)	4.732 (1.4-15.8)
First response to general health problem			
Consult physician /health-worker	57 (56.4)	24 (24.0)	Reference
Home remedy/Herbal medicines	27 (26.7)	44 (44.0)	3.870(2.0-7.6)
Medicines from store	17 (16.8)	32 (32.0)	4.471(2.1-9.5)
Previous experience with cancer			
Yes	39 (32.2)	24 (17.0)	Reference
No	81 (66.9)	117 (83.0)	2.377(1.3-4.3)
Travel options to nearest healthcare facility			
Single vehicle transport	89 (74.2)	83 (58.9)	Reference
Multiple vehicle transport	31 (25.8)	58 (41.1)	2.006(1.2-3.4)
Distance to nearest healthcare facility			
≤ 3 kilometres	58 (48.3)	41 (29.1)	Reference
Above 3 kilometres	62 (51.7)	100(70. 9)	2.282(1.4-3.8)
Time to reach nearest healthcare facility			
≤ 10 minutes	36 (30.0)	22 (15.6)	Reference
11 – 30 minutes	72 (60.0)	83 (58.9)	1.886 (1.0- 3.5)
31 minutes and above	12 (10.0)	36 (25.5)	4.909(2.1-11.4)
Current problem in oral cavity			
Discussed present problem in oral cavity with someone before meeting HCP			
Yes	99 (82.5)	81 (57.4)	Reference
No	21 (17.5)	60 (42.6)	3.492 (1.96-6.2)
Reason for meeting HCP for current problem in oral cavity			
Pain	36 (30.0)	62 (44.0)	11.19 (4.3-29.0)
Discomfort to daily routine	45 (37.5)	73 (51.8)	10.54 (4.1- 26.9)
Insistence by family/friends	39 (32.5)	6 ( 4. 3)	Reference
Cancer stage			
Early (Stage 1&2)	60 (50.0)	23 (16.3)	Reference
Late (Stage 3&4)	60 (50.0)	118 (83.7)	5.130 (2.9-9.1)
First response to the current problem in oral cavity			
Ignored the symptoms	54 (45.0)	80 (56.7)	Reference
Tried local remedies	27 (22.5)	53 (37.6)	1.325 (0.7-2.4)
Consulted Doctor	39 (32.5)	8 (5.7)	0.138 (0.06-0.32)

OR, Odds ratio; HCP, Healthcare Personnel

**Table 3:** Endorsement of Barriers to Help-Seeking and Patient Interval in Oral Cancer Patients (n= 261)

Variable	≤ 90 days n (%)	> 90 days n (%)	OR (95%CI)
Found it embarrassing talking to the doctor about symptoms			
Yes	21 (17.5)	46 (32.6)	2.283(1.3-4.1)
No	99 (82.5)	95 (67.4)	Reference
Was too busy to make time to go to the doctor			
Yes	40 (33.3)	68 (48.2)	1.863(1.1-3.1)
No	80 (66.7)	73 (51.8)	Reference
Had too many other things to worry about			
Yes	49 (40.8)	82 (58.2)	2.014 (1.2-3.3)
No	71 (59.2)	59 (41.8)	Reference
Was worried about what tests they might want to do			
Yes	29 (24.2)	70 (49.6)	3.094 (1.8-5.3)
No	91 (75.8)	71 (50.4)	Reference
Did not have a person to accompany me during hospital visits/consultations			
Yes	20 (16.7)	44 (31.2)	2.268 (1.2-4.1)
No	100 (83.3)	97 (68.8)	Reference
Did not have money to consult a doctor/visit a hospital			
Yes	23 (19.2)	47 (33.3)	2.109 (1.2-3.7)
No	97 (80.8)	94 (66.7)	Reference
Was worried about what the doctor might find out			
Yes	23 (19.2)	64 (45.4)	3.505 (2.0-6.2)
No	97 (80.8)	77 (54.6)	Reference
Comfortable in discussing symptoms with a nurse than a doctor			
Yes	32 (26.7)	55 (39.0)	1.759 (1.03-3.0)
No	88 (73.3)	86 (61.0)	Reference
Prefer alternative medicines than modern medicine			
Yes	17 (14.2)	42 (29.8)	2.570 (1.4-4.8)
No	103 (85.8)	99 (70.2)	Reference

**Table 4:** Summary of Binary Logistic Regression Analysis for Variables Predicting Patient interval

Variable	Patient interval n (%) ≤90 days	Patient interval n (%) > 90 days	Adjusted Odds ratio (95% CI)
Reason for meeting HCP for current problem in oral cavity			
Pain	36 (30)	62 (44)	8.300 (2.9-23.4)
Discomfort to daily routine	45 (38)	73 (52)	6.982 (2.5-19.3)
Insistence by family/friends	39 (33)	6 ( 4)	Reference
Tobacco smoking status at the time of symptom recognition			
Current smoker	38 (32)	63 (45)	2.518 (1.3-4.7)
Former smoker	19 (16)	11 ( 8)	0.717 (0.3-1.9)
Never smoker	63 (53)	67 (48)	Reference
Cancer stage			
Early (Stage 1&2)	60 (50)	23 (16)	Reference
Late (Stage 3&4)	60 (50)	118 (84)	2.621(1.3-5.2)
Was worried about what the doctor might find out			
Yes	23 (19)	64 (45)	2.546 (1.3-4.9)
No	97 (81)	77 (55)	Reference
Time to reach nearest healthcare facility			
≤ 10 minutes	36 (30)	22 (16)	Reference
11 – 30 minutes	72 (60)	83 (59)	1.443 (0.6- 3.4)
31 and above	12 (10)	36 (26)	5.803 (1.6-21.7)

Note: Control was ‘Distance to nearest healthcare facility’(omitted from the table)

## Discussion

To our knowledge, this is the first study on the patient interval in oral cancer from Kerala, India designed and conducted as per the Aarhus statement. Early diagnosis reduces morbidity and mortality associated with oral cancer (van der Waal, 2013). The time took by the patients for self-referral when they notice those symptoms suggestive of cancer is a key factor in the diagnostic pathway. The median patient interval of 92 days reported in our study is in agreement with a similar observation from a regional cancer center in northeast India (Baishya et al., 2015), but there are reports of median patient interval ranging from 35 days (Jovanovic et al., 1992) to 123 days (Peacock et al., 2008) in other studies. A study from a tertiary care hospital in north India had reported increased patient interval in 60%of their study participants (Akram et al., 2014) whereas, in our study, this proportion was 54%. These findings point to the higher prevalence of increased patient interval in our study population.

We took every effort to conduct and report our study in concurrence with “Aarhus statement” (Weller et al., 2012). The beginning and endpoints of patient interval duration were clearly defined before the initiation of data collection using a validated instrument (Philip and Kannan, 2019). At the outset, the present study findings confirm the fact that oral cancer is a disease of the elderly. Studies from some western countries had reported mean age as high as 76 years (Goldenberg et al., 2014). This is true with oral precancerous lesions also (Pindborg, 1978). Though the relationship between age and occurrence of oral cancer is well established, we couldn’t identify any such relationship between age and patient interval. The previous studies on patient interval had given contradicting results on socio-demographic factors that contribute to the prolonged patient interval (Philip and Kannan, 2019). The lack of association of other socio-demographic factors with patient interval may be due to the known relationship between oral cancer and low socioeconomic status (Madani et al., 2010).

The type of house in which the patient lived was the only socio-demographic factor found to be significantly associated with the patient interval in our study. A study from south India reports a higher prevalence of tobacco smoking among those who live in semi-pucca-type houses in comparison to those living in pucca-type houses (Vinothkumar et al., 2020). Less affluent households often have poor housing conditions and that may affect health outcomes (Braubach and Savelsberg, 2009). This finding supports our observation that a higher proportion of participants with the patient interval of more than 90 days had semi-pucca-type houses and were ever tobacco users unlike those with the pucca house. 

Factors linked to the pattern of health-seeking like self-remedy, self-medication, and consulting traditional healers were found to delay symptom presentation in a study from a developing country (Azhar and Doss, 2018). We also had similar observations in our study. Those who were reluctant to visit medical/dental facilities were at higher risk of having increased patient interval. High-risk people seldom visit a dentist or dental screening programs (Peacock et al., 2008). In our study, only 5.4% of participants reported a history of dental screening. Access to the health facility was an important determinant of patient interval duration. A study from Sri Lanka identified the cost of travel as a factor associated with patient interval (Alahapperuma and Fernando, 2017). A systematic review on the relationship of travel distance and travel duration on health outcomes in global north countries had found that the majority of the included studies report worse health outcomes among those who lived farther from the health facility (Kelly et al., 2016). In our study, we examined those disease factors that were directly linked to the current disease and found the stage of disease as a significant predictor of increased patient interval. A Finnish study on patient delay among oropharyngeal cancer patients also reported the association of cancer stage with patient interval (Nieminen et al., 2020). The stage of cancer is an important predictor for prognosis and survival in oral cancer (Thavarool et al., 2019). Thus any reduction in the patient interval duration will help to add quality years to the patient’s life. Oral cancer in the initial stages may be asymptomatic or have symptoms that appear to be less severe or alarming and hence people ignore them. The pain usually presents in the late stages. Hence those who meet a doctor only when they have persistent pain or discomfort had increased patient interval. 

We found that sharing symptoms with friends or family and taking them into confidence will reduce the patient interval. Discussing symptoms with family or friends may act as a trigger for presenting symptoms suggestive of cancer immediately to a health care provider. It is of concern that a notable proportion (31%) of participants with oral cancer had failed to discuss their symptoms. The study by Rogers et al., (2011) supports this observation. This indicates the need for providing cancer symptom awareness education in families and workplaces, especially in high-risk communities. In our study, 82% of participants either ignored their symptoms or tried local remedies resulting in the postponement of medical consultation and prolongation of patient interval. In one study, nearly half of the oral cancer patients considered their symptoms as not serious and another one-third tried local remedies instead of seeking professional help. These findings provide scope for interventions that focus on oral cancer symptom awareness education. These interventions should highlight the importance of early presentation for better prognosis and survival. The triggering reason for meeting the HCPs was mainly symptom-related discomfort or pain (83%) and a higher proportion of these participants were in advanced stages in comparison with those reported due to insistence by friends or family and this association was statistically significant. This suggests that people should not wait for oral symptoms to get aggravated for reporting. Symptoms generally get aggravated in late stages. Care should be taken to report any symptoms, however mild it may be to a health care provider irrespective of its perceived severity. 

In conclusion, health education on symptom appraisal and familiarisation of health care facilities in the context of cancer detection may improve symptom reporting at the primary care level. Most of the oral cancer patients are working in the unorganized sector where provisions for availing medical care were non-existent. Work site-based health education and early diagnosis interventions should be planned and provisions for periodic free health check-ups should be made mandatory. The double burden of tobacco use in oral cancer, as it increases the risk of disease occurrence and delays symptom presentation, needs serious policy considerations in the context of cancer prevention. Man, being a social animal, is influenced by the people around him. Sharing health-related concerns with the people around him may speed up the diagnostic journey. People at high risk of developing oral cancer should be given health education on the warning signs and curability of early-stage oral cancer. Although oral cancer is one of the few cancers that have the ideal characteristics suitable for early detection, most of the cases are reported in advanced stages. One of the solutions for reducing late-stage reporting is to decrease the patient interval. The patient interval can be shortened by addressing the relevant individual, interpersonal and organizational factors like symptom awareness, habit patterns, health-seeking and help-seeking attitudes, family and social support, and accessibility and availability of health care facilities. 

## Author Contribution Statement

Conceptualization: PMP, SK; Design: PMP, SK; Literature search: PMP; Data acquisition: PMP; Data analysis: PMP, SK; Manuscript preparation: PMP; Manuscript editing: PMP, SK; Manuscript review: SK.
